# Fungal Deoxynivalenol-Induced Enterocyte Distress Is Attenuated by Adulterated Adlay: *In Vitro* Evidences for Mucoactive Counteraction

**DOI:** 10.3389/fimmu.2018.00186

**Published:** 2018-02-23

**Authors:** Zhimin Du, Ki Hyung Kim, Juil Kim, Yuseok Moon

**Affiliations:** ^1^Laboratory of Mucosal Exposome and Biomodulation, Department of Biomedical Sciences and Biomedical Research Institute, Pusan National University, Yangsan, South Korea; ^2^Department of Obstetrics and Gynecology, Pusan National University School of Medicine, Pusan, South Korea; ^3^Program of Intelligent Food Health Sciences and Institute of Marine Biotechnology, Pusan National University, Busan, South Korea

**Keywords:** adlay, gut barrier, deoxynivalenol, inflammation, wound

## Abstract

Adlay is a cereal crop that has long been used as traditional herbal medicine and as a highly nourishing food. However, deoxynivalenol (DON), the most prevalent trichothecene mycotoxin worldwide, frequently spoils grains, including adlay, *via* fungal infection. On the basis of an assumption that the actions of DON in the gut could be modified by adlay consumption, we simulated the impacts of co-exposure in enterocytes and investigated the effectiveness of treatment with adlay for reducing the risk of DON-induced inflammation and epithelia barrier injury. In particular, adlay suppressed DON-induced pro-inflammatory signals such as mitogen-activated kinase transduction and the epidermal growth factor receptor-linked pathway. In addition to regulation of pro-inflammatory responses, adlay treatment interfered with DON-induced disruption of the epithelial barrier. Mechanistically, adlay could boost the activation of protein kinase C (PKC) and cytosolic translocation of human antigen R (HuR) protein, which played critical roles in the epithelial restitution, resulting in protection against disruption of enterocyte barrier integrity. Notably, DON abrogated the Ras homolog gene family member A GTPase-mediated actin cytoskeletal network, which was diminished by adlay treatment in PKC and HuR-dependent ways. Taken together, this study provides evidences for adlay-based attenuation of trichothecene-induced gut distress, implicating potential use of a new gut protector against enteropathogenic insults in diets.

## Introduction

Among the broadly used herbal medicines for gut health, cereal-based adlay (Job’s tears, *Coix lachryma-jobi* L.) is a monocotyledon-based grain-bearing crop that is widely planted in many Asian countries. However, herbal medicines can be contaminated by a broad variety of fungal toxic metabolites, including mycotoxins from *Aspergillus* spp., *Penlicillium* spp., or *Fusarium* spp (1–3). Naturally occurring mycotoxins limit the utilization of medicinal materials and introduce potential risks to human and animal health. *Fusarium* head blight and its toxic metabolites such trichothecenes are present in adlay (family: Poaceae) as a rich source of *Fusarium* species (4, 5). The trichothecene mycotoxins, a group of secondary metabolites produced by sesquiterpenoid fungi, are widely found with high prevalence in cereals, grains, feedstuffs, and indoor air (6). Among the trichothecenes, deoxynivalenol (DON; vomitoxin) is mainly produced by *Fusarium graminearum* and *Fusarium culmorum* (7, 8), which colonize more than 60–70% of world agricultural commodities, including a variety of cereal-based feed and foodstuffs (9). DON also frequently spoils grains with medicinal properties, such as adlay, *via* infection with the mold (5).

Epidemiological studies suggest links between exposure to trichothecene mycotoxins, including DON and gastrointestinal illness in human and animals (10–12). Exposure to trichothecene mycotoxins and their metabolites in experimental models also leads to mucosal inflammatory and oxidative injuries in the gastrointestinal tracts and airways (13–19). In addition to excessive activation of the immune system, DON may alter the gut epithelial barrier, facilitating gut microbial translocation and subsequent submucosal pro-inflammatory activation (14, 20, 21). At the cellular level, DON impacts highly proliferating tissues in the gastrointestinal tracts and the immune system (22). A variety of fungal trichothecene metabolites can damage the functionality of the 28S ribosomal RNA during gene translation, leading to a ribotoxic stress response that stimulates intracellular sentinel signaling pathways, including the mitogen-activated protein kinase (MAPK) as the central signal. This process results in the expression of genes important for cellular homeostasis, as well as genes integral to a variety of immunopathogenic processes involved in cell survival, inflammation, and stress responses (23, 24).

Although DON in itself insults the gut and other susceptible organs, the ultimate dietary toxicity would be altered by interaction with food. Depending on the presence of bioactive food components, the net toxicity of DON in the food matrix could be either attenuated or incremented. In particular, contamination with trichothecene mycotoxins including DON in adlay grains frequently occurs because of improperly practiced postharvest procedures (5). Adlay grains and their components have been shown to possess anticancer (25), antioxidant (26, 27), antiallergic (28), and anti-inflammatory activities (29–31). On the basis of assumption that gastrointestinal actions of DON could be affected by adlay consumption, we simulated the impacts of co-exposure to DON and adlay using an enterocyte culture model and investigated the detailed molecular mechanisms of nutritional crosstalk in the gastrointestinal barrier, the early frontline of defense against xenobiotics and pathogens.

## Materials and Methods

### Cell Culture and Reagents

Intestinal epithelial cells (IECs) including the colorectal adenocarcinoma-derived enterocytes (HT-29, HCT-8, LS174T, DLD-1, and SW480) and the well-established non-tumorigenic normal enterocytes from the porcine gut (IPEC-1) were purchased from the American Type Culture Collection (Manassas, VA, USA) and Leibniz-Institute DSMZ (Brauschweig, Germany), respectively. In this study, HT-29 cells were used as a representative model of pathogenesis in the IECs because they show differentiated and polarized features in RPMI 1640-based media (32). In addition, HCT-8 or IPEC-1 cell-based barrier was assessed due to its rapid formation of epithelial monolayer among the colorectal adenocarcinoma-derived enterocytes. In particular, IPEC-1 cells have been extensively used as the non-tumorigenic enterocyte model of DON exposure (17, 33, 34). The colorectal adenocarcinoma-derived enterocytes were cultured in RPMI 1640 medium supplemented with 10% heat-inactivated FBS, 50 U/ml penicillin, and 50 µg/ml streptomycin (all from Welgene, Daegu, South Korea) in a 5% CO_2_ humidified incubator at 37°C. Since RPMI 1640 media in this study do not contain HEPES buffer, 25 mM HEPES (LPS Solution, Daejeon, South Korea) was additionally administered in LS174T and SW480 cell culture for optimal buffering capacity. Other colon adenocarcinoma-derived cells optimally grow in HEPES-free RPMI-based media. IPEC-1 cells were maintained in DMEM/F12 (Welgene, Daegu, South Korea) supplemented with 10% (v/v) heat-inactivated FBS, 50 units/ml penicillin, and 50 µg/ml streptomycin in a 5% CO_2_ humidified incubator at 37°C. The viable cells determined by staining with trypan blue (Sigma-Aldrich, St. Louis, MO, USA) were counted using a hemocytometer. Mycotoxin-free adlay was kindly provided by Rural Development Administration, Korea. SB203580 and U0126 were purchased from Calbiochem (Merck Millipore, Billerica, MA, USA) and Enzo Life Science (Plymouth, PA, USA), respectively. All other chemicals were purchased from Sigma-Aldrich.

The ethyl acetate fraction of adlay bran was extracted because this fraction includes high levels of anti-inflammatory components such as chlorogenic acid, vanillic acid, caffeic acid, 4-hydroxyacetophenone, p-coumaric acid, syringaldehyde, ferulic acid, and 6-methoxy-2-benzoxazolinine as previously reported (29, 35). Briefly, dried adlay bran was extracted three times with 95% ethanol (200 ml each time) using a stirring apparatus in a brown bottle. The combined extracts were then passed through No. 1 Whatman filter paper, after which the filtrate was evaporated under vacuum at 38°C to obtain a residue, which was subsequently suspended in water and then successively partitioned with hexane (1:1) and ethyl acetate (1:1). This produced an ethyl acetate-soluble fraction, which was evaporated again and dissolved in DMSO.

### Plasmid Construction

Interleukin-8 (IL-8) transcriptional activity was measured using IL-8 promoter-luciferase reporter construct (36–38). IL-8 promoter-luciferase construct containing a part of the human IL-8 promoter ranging from nucleotides −416 to +44 was kindly provided from Dr. Kung, Hsing-Jien (University of California at Davis). The 3′ untranslated region (UTR) of the human IL-8 gene (+402/+1611) was cloned into the pGL3 control vector at the XbaI site. After polymerase chain reaction (PCR) of the promoter region with Pfu turbo DNA polymerase (Stratagene, La Jolla, CA, USA), the fragment was cloned into the TA vector (Invitrogen), sequenced, and subcloned into the pGL3 control vector. An antisense (AS) human antigen R (HuR) gene construct was kindly provided from Dr. Gorospe, Myriam (NIH, Baltimore, USA) and reconstituted into pcDNA3.1-Hyg vector system (Invitrogen). The final product was designated pcDNA3.1-AsHuR-Hyg (HuR-AS). CMV-driven small interference RNA (siRNA) expression vector was constructed by inserting the hairpin siRNA template into a pSilencer 4.1-CMV-neovector (Ambion Inc., Austin, TX, USA). The scrambled control vector and early growth response gene 1 (Egr1) siRNA insert-containing vector were denoted as pSilencer and pSiEgr1, respectively. Insert Egr1 siRNA (Dharmacon, Lafayette, CO, USA) targeted the sequence AAGTTACTACCTC TTATCCAT.

### Cell Proliferation Assay

HT-29 cells (6 × 10^3^) were seeded in a 96-well culture plate in 6 replicates, cultured for 48 h, and then treated with the vehicle or each combination of chemicals for indicated times. At the end of treatment, 50 µl 3-(4,5-dimethylthiazol-2-yl)-2,5-diphenyl tetrazolium bromide (1 mg/ml) was added to each well (100 µl media), and the plates were incubated at 37°C for 4 h to measure its reduced metabolite (formazan crystal) *via* mitochondrial action (39). The formazan product was then dissolved in 150 µl DMSO at 37°C for 30 min, and the absorbance at 540 nm was measured with a microplate reader.

### RT-PCR and Real-time Quantitative PCR

HT-29 cells (3 × 10^5^), HCT-8 cells (3 × 10^5^), DLD-1 cells (3 × 10^5^), LS174T cells (3 × 10^5^), SW480 cell (3 × 10^5^), and IPEC-1 (2 × 10^5^) were seeded, cultured for 48 h, and then treated with the vehicle (DMSO) or each dose of DON with or without adlay extract. Cellular RNA was extracted using RiboEX (GeneAll Biotech, Seoul, South Korea), and the subsequent procedure was performed based on the methods reported in our previous study (38). The mRNA was transcribed into cDNA by Prime RT premix (Genet Bio, Nonsan, South Korea). cDNA amplification was conducted with N-Taq DNA polymerase (Enzynomics, Seoul, South Korea) in a MyCycle thermal cycler (BioRad) using the following parameters: initial denaturation at 95°C for 2 min, followed by varying numbers of cycles of denaturation at 95°C for 30 s, annealing at 58°C for 30 s, and elongation at 72°C for 30 s. An aliquot of each PCR product was subjected to 1.2% (w/v) agarose gel electrophoresis and visualized by ethidium bromide staining. Sequences of PCR primers for amplifying each gene were as follows: human GAPDH (5′-TCA ACG GAT TTG GTCGTA TT-3′ and 5′-CTGTGG TCA TGA GTC CTT CC-3′), human Egr1 (5′-AGC ACC TGA CCG CAG AGT CT-3′and 5′-AGA TGG TGC TGA GGA CGA GG-3′), human IL-8 (5′-ATG ACT TCC AAG CTG GCC GTG GCT-3′5′-TCT CAG CCC TCT TCA AAA ACT TCT C-3′), porcine IL-8 (5′-ACT TCC AAA CTG GCT GTT GC-3′ and 5′-TGC TGT TGT TGT TGC TTC TCA-3′), and porcine GAPDH (5′-CAC GAC CAT GGA GAA GGC-3′ and 5′-GAA GCA GGG ATG ATG TTC TGG-3′). Real time PCR was conducted using an iCycler Thermal cycler (BioRad) with the following parameters: initial denaturation at 95°C for 15 min followed by cycles of denaturation at 95°C for 20 s, annealing at 59°C for 30 s, and elongation at 72°C for 30 s. Each sample was assessed in triplicate, after which the relative quantification of gene expression was performed by the comparative threshold cycle (C_t_) method. GAPDH was used as the endogenous control, and each independent experiment was repeated three times.

### Enzyme-Linked Immunosorbent Assay (ELISA)

The IL-8 concentrations in cell culture supernatants were determined using a commercially available ELISA kit (BD Biosciences, Franklin Lakes, NJ, USA) according to the manufacturer’s directions. HT-29 cells were seeded at 1 × 10^5^ cells/well of a 24-well plate (*n* = 6) and cultured for 48 h. After treatment with the vehicle (DMSO) or each combination of chemicals for 24 h, cell culture medium was collected, and cell debris was removed by centrifugation. Briefly, the capture antibody was coated onto the wells of ELISA plates overnight at 4°C. After washing with Tween 20-containing PBS and blocking with PBS supplemented with 10% (v/v) FBS overnight at 4°C, the plates were incubated with serial dilutions of IL-8 samples and standards. After incubation with the detection antibody and the tetramethylbenzidine substrate, the absorbance was measured at 450 nm using an ELISA reader, and the assay detection limit was 3.1 pg/ml of IL-8 (38). Results are representative of two independent experiments.

### Western Blot Assay

HT-29 cells (5 × 10^5^), DLD-1 cells (5 × 10^5^), LS174T cells (5 × 10^5^), SW480 cell (5 × 10^5^), and IPEC-1 (4 × 10^5^) were seeded, cultured for 48 h, and then treated with the vehicle (DMSO) or each combination of chemicals for 0.5, 1, or 2 h. Treated cells were washed with ice-cold phosphate buffer, lysed in boiling lysis buffer [1% (w/v) SDS, 1.0 mM sodium orthovanadate, and 10 mM Tris, PH 7.4] and sonicated for 5 s. The proteins in these lysates were then quantified using a BCA protein assay kit (Pierce, Rockford, IL, USA). Equal amounts of protein (30 µg) were separated by SDS-PAGE gel in a BioRad gel mini electrophoresis system (BioRad, Hercules, CA, USA). The proteins were transferred onto a PVDF membrane (Pall Corporation, New York, NY, USA) and then blocked for 1 h with 5% skim milk in tris-buffered saline plus 0.1% Tween (TBST), after which they were incubated with the desired primary antibody overnight at 4°C. After washing three times with TBST, the blots were incubated with horseradish-conjugated secondary antibody for 2 h and then washed with TBST three times (40). Antibody binding was detected with an ECL substrate (ELPIS Biotech, Daejon, South Korea). The following antibodies were used for Western blot: rabbit polyclonal anti-Actin, mouse monoclonal anti-β-tubulin, rabbit polyclonal anti-p-p38, mouse monoclonal anti-p-Erk, rabbit polyclonal anti-Egr1, mouse monoclonal anti-hnRNP, mouse monoclonal anti-HuR (Santa Cruz Biotechnology, Santa Cruz, CA, USA), rabbit polyclonal p-(Ser) protein kinase C (PKC) Substrate Antibody, and rabbit anti-p-EGF Receptor (Y1068) (Cell Signaling Technology, Beverly, MA, USA).

### Isolation of Cytosolic and Nuclear Extracts for Immunoprecipitation (IP) Assay

HT-29 cells (7 × 10^5^) were seeded, cultured for 48 h, and then treated with the vehicle (DMSO) or each combination of DON and adlay for 1 h. Cells were harvested from culture plates by scraping in ice-cold PBS and centrifuged at 200 × *g* once for 3 min. The cell pellet after centrifugation was then resuspended in a lysis buffer containing 10 mM HEPES, 10 mM KCl, 1.5 mM MgCl_2_, 0.5 mM DTT, 0.5 mM PMSF, 0.1% Nonidet P-40, and protease inhibitor mixture (Sigma-Aldrich), incubated for 10 min on ice, and centrifuged at 900 × *g* once for 15 min. Next, the supernatant (cytosolic fraction) was collected, and the remaining pellet was resuspended in a buffer containing 20 mM HEPES, 1.5 mM MgCl_2_, 420 mM NaCl, 0.2 mM EDTA, 0.5 mM DTT, 0.2 mM PMSF, 25% glycerol, and protease inhibitor mixture (Roche). After 10 min of incubation on ice, samples were centrifuged at 13,800 × *g* once for 30 min, and the supernatants (nuclear proteins) were collected, aliquots, and stored at −80°C before analysis. Next, rabbit polyclonal anti-p-PKC and mouse monoclonal anti-HuR were added to the cell cytosolic fraction lysate supernatant and rotated overnight at 4°C. Protein G-Sepharose (30 µl; Santa Cruz Biotechnology) in antibody-cell lysate were incubated by rotating at 4°C for 3 h. The antibody cytosolic fraction extracts were then washed three times with IP lysis buffer (50 mM Tris, pH 7.2, 150 mM NaCl, 1 mM EDTA, 400 mM Na_3_VO_4_, and 2.5 mM phenylmethanesulfonyl fluoride), after which 6× SDS sample buffer (60% glycerol, 300 mM Tris pH 6.8, 12 mM EDTA, 12% SDS, 864 mM 2-ME, and 0.05% bromophenol blue) was added. Finally, immunoprecipitates were collected by centrifugation and subjected to SDS-PAGE. The nuclear (hnRNP) and cytoplasmic (β-actin) markers verified the identity and purity of the fractions (38).

### Transfection and Luciferase Assay

HT-29 cells (2.5 × 10^5^) were seeded, cultured for 48 h, and then transfected with a shRNA vector using OmicsFect (Omics Biotechnology Co., Taipei City, Taiwan), jetRPIME (polyplus transfection), or Lipofector-EXTReagent (Aptabio Therapeutics Inc., Gyeonggi-do, Korea) according to the manufacturer’s protocol. For transfection of the luciferase reporter gene, a mixture of 1 µg of luciferase reporter plasmids and 0.1 µg of Renilla luciferase pRL-null vector (Promega, Madison, WI, USA) per 1 µl of Carrigene (Kinovate, Seoul, Korea) was applied to wells of a 12-well culture plate. Transfected cells were exposed to vehicle (DMSO) or each combination of chemicals for 12 h and lysed for luciferase assay. All transfection efficiency was maintained at around 50–60%, which was confirmed with pMX-enhanced green fluorescent protein vector. Cells were washed with cold PBS and then lysed with passive lysis buffer (Promega, Madison, WI, USA). Cell lysates were centrifuged, and the supernatant was collected for luciferase activity, which was measured with a dual-mode luminometer (Model TD-20/20, Turner Designs Co., Sunnyvale, CA, USA) after briefly mixing 10 µl supernatant and 50 µl firefly luciferase assay substrate solution, followed by 50 µl stopping Renilla luciferase assay solution (Promega, Madison, WI, USA). The firefly luciferase activity was normalized against Renilla luciferase activity using the following formula: firefly luciferase activity/Renilla luciferase activity (41). This experiment was conducted in triplicate, and all of the results are representative of three independent experiments.

### Confocal Microscopy

HT-29 cells (1 × 10^5^) or HCT-8 cells (1 × 10^5^) were cultured in glass-bottom culture dishes (SPL Life Science, Pocheon, Korea) and cultured for 48 h. Following treatment with the vehicle (DMSO) or each combination of chemicals for 1 or 24 h, cells were fixed with 4% paraformaldehyde (Biosesang, Sungnam, Korea) and permeabilized with 0.2% Tween 20 and 0.3% BSA in PBS for 10 min. After 2 h of blocking with 3% BSA in PBS, cells were incubated with primary antibody in 3% BSA PBS for 2 h at room temperature. Subsequently, the cells were washed with PBS and incubated with goat anti-mouse IgG (H + L) Dylight 488 conjugated secondary antibody (Bethyl Laboratories, Montgomery, TX, USA) or Texas Red (SurModics, Eden Prairie, MN, USA) for 2 h at room temperature. The cells were repeatedly washed in PBS and stained with 100 ng/ml DAPI in PBS for 10 min (41). Next, confocal images were obtained using an Olympus FV1000 confocal microscope (Olympus, Tokyo, Japan). The images were acquired and processed with FV10-ASW software (Olympus, Tokyo, Japan), and the intensity of signals from individual cells was measured using the ImageJ 1.45 software. This experiment was conducted in triplicate, and all of the results are representative of three independent experiments.

### Measurement of Transepithelial Electrical Resistance (TEER)

Cells (HCT-8 or IPEC-1) were seeded at a density of 4 × 10^5^ cells per well and grown into monolayers in 24-well transwell filters with 0.4-µm pores (Becton-Dickinson Labware, Franklin Lakes, NJ, USA). For the cell differentiation, cell culture media containing 100 nM dexamethasone was added and exchanged every 2 days until complete differentiation (42). On 10th day of the differentiation process, cells were treated with the vehicle (DMSO) or each combination of chemicals. TEER was measured every 12 h with an EVOM2 epithelial voltohmmeter (World Precision Instruments, Sarasota, FL, USA). Experimental TEER values were expressed as Ω cm^2^. This experiment was conducted in triplicate, and all of the results are representative of three independent experiments.

### Paracellular Tracer Flux Assay

Fluorescein isothiocyanate (FITC)–dextran was used as a fluorescent probe to study cell permeability. Briefly, cells were seeded at a density of 4 × 10^5^ cells per well and grown into monolayers in 24-well transwell filters with 0.4 µm pores, and differentiated cells in the transwell inserts were treated with the vehicle (DMSO) or each combination of chemicals for 48 h. Next, FITC–dextran (Sigma-Aldrich Chemical Company) dissolved in cell culture media was added to the apical compartment at a final concentration of 2.2 mg/ml. After 1 h of incubation, the intensity of fluorescence emission in the outer part of the well was measured using a Victor 3 fluorometer (Perkin Elmer, Waltham, MA, USA), with excitation and emission wavelengths set to 490 and 535 nm, respectively (33). This experiment was conducted in triplicate, and all of the results are representative of three independent experiments.

### Wound Healing Assay

For two-sided wound healing assays, cells (HCT-8 or IPEC-1) were seeded to a final density of 5 × 10^5^ cell/ml in an Ibidi Culture-Insert 2 well (Ibidi, Martinsried, Germany) according to the manufacturer’s directions. After cell attachment, the Ibidi Culture-Insert was gently removed creating a gap of ~500 μm. Cells were gently washed with PBS, and the dish was filled with serum-free medium containing the vehicle (DMSO) or each combination of chemicals. Images of the cells migrating into the wound were captured by an inverted microscope (×10; *n* = 3). The migration was measured by calculating mean of distances of the migrating cells from both wound edges using JR screen ruler. For the IPEC-1 cells, the relative area of migrating cells in the wound space was measured using the ImageJ 1.45 software since the migrating edge was not linear. For scratch-induced wound healing assays, cells were seeded in 12-well culture plates and grown to a monolayer. Because of irregular gap after scratching, one-sided migration was quantified. After scratching, cells were incubated with serum-free medium. Images of the cells migrating into the wound were captured by an inverted microscope (×10; *n* = 3–6). The average wound length was measured by calculating mean of distances of the migrating cells from one side of wound edges using JR screen ruler. This experiment was conducted in triplicate, and all of the results are representative of three independent experiments.

### Statistical Analysis

Statistical analysis was performed using the GraphPad Prism 6 software (GraphPad Software, La Jolla, CA, USA). A Student’s *t*-test was used for comparative analysis of the two groups of data. To compare multiple groups, data were subjected to ANOVA, and pairwise comparisons were made by the Student–Newman–Keuls method.

## Results

### Adlay Extract Downregulates DON-Induced Production of IL-8 in Gut Epithelial Cells

**Figure 1:**
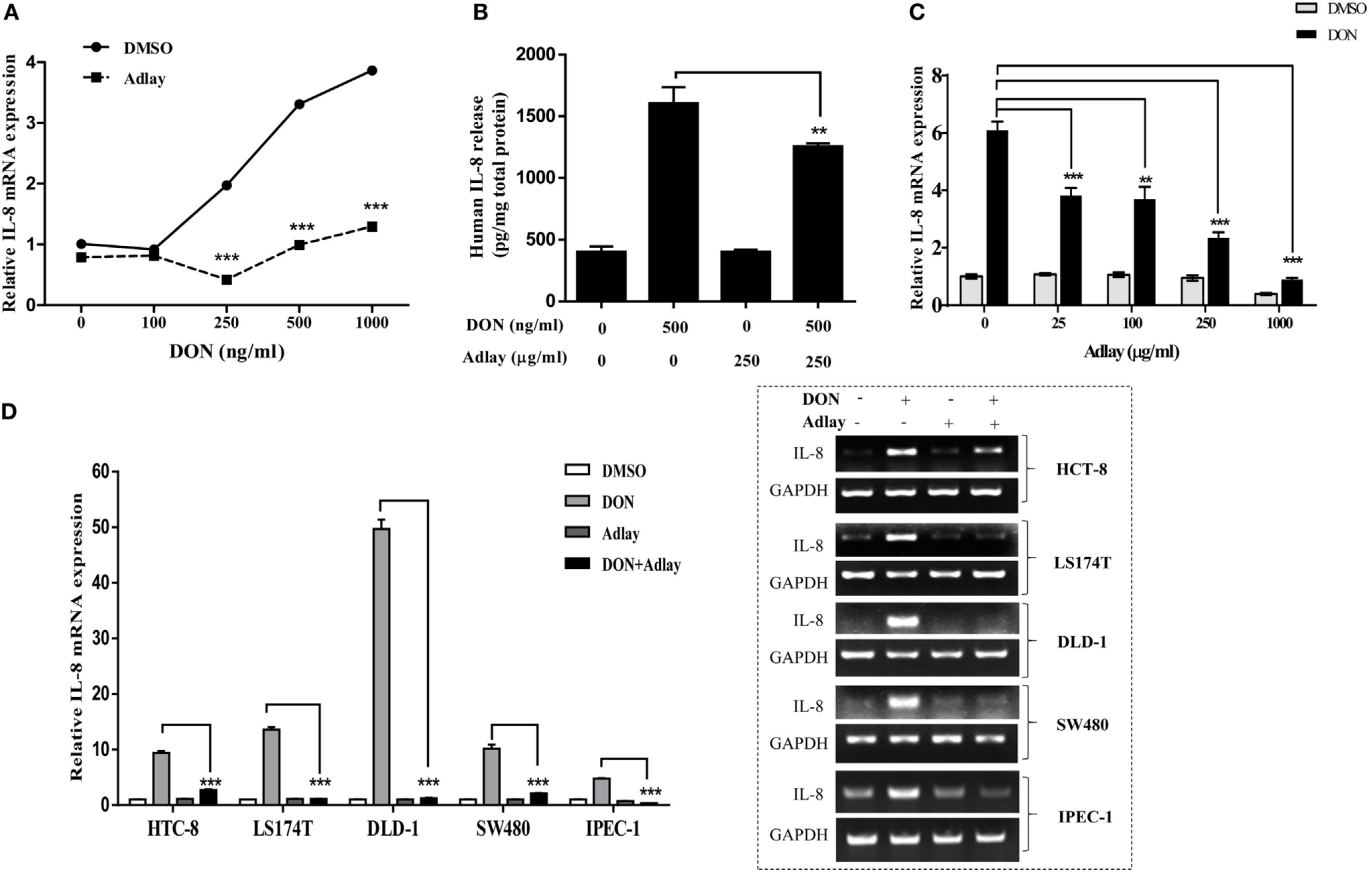
Adlay bran suppresses deoxynivalenol (DON)-induced chemokine production in enterocytes. **(A)** HT-29 cells were treated with the indicated dose (0, 100, 250, 500, or 1000 ng/ml) of DON in the absence or presence of 1,000 µg/ml adlay bran extract for 1 h, after which interleukin (IL)-8 expression was measured using real-time quantitative polymerase chain reaction (qRT-PCR; *n* = 3, ***p < 0.001, significant difference from DMSO-treated group at each dose of DON). **(B)** IL-8 secretion was analyzed by enzyme-linked immunosorbent assay of the culture media of HT-29 cells co-treated with 500 ng/ml DON and the indicated dose (0 or 250 µg/ml) of adlay bran extract for 24 h (*n* = 6, **p < 0.01, significant difference from DON-treated group without adlay). **(C)** HT-29 cells were treated with 500 ng/ml DON in the absence or presence of 0, 25, 100, 250, or 1,000 µg/ml adlay bran extract for 1 h, after which mRNA expression was quantified using qRT-PCR (*n* = 3, **p < 0.01, ***p < 0.001, significant difference from DON-treated group without adlay). **(D)** HCT-8, LS174T, DLD-1, SW480, or IPEC-1 cells were treated with 500 ng/ml DON in the absence or presence of 1,000 µg/ml adlay bran extract for 1 h, after which IL-8 expression was measured using qRT-PCR (left) and conventional RT-PCR (the right box) (*n* = 3, ***p < 0.001, significant difference from DON-treated group without adlay). All of the results are representative of three independent experiments.

Mucosal epithelium senses external toxic insults and transmits danger signals to activate a broad range of defensive inflammatory responses, including chemokine production. Adlay-treated HT-29 cells showed attenuated cellular induction of IL-8 in response to DON in a dose-dependent manner (Figure 1A). DON-triggered IL-8 release was thus reduced by adlay treatment (Figure 1B). Suppression of IL-8 mRNA was also demonstrated in enterocytes treated with lower levels of adlay extract (Figure 1C). In the early treatment (1–2 h) for the gene expression analysis, the cell growth was not significantly suppressed by DON or adlay (Figure S1A in Supplementary Material). However, extended exposure (24 h) to higher doses (>500 µg/ml) of adlay extract reduced the cell growth in presence or absence of DON (Figure S1B in Supplementary Material). Therefore, all biochemical readouts were divided by the total levels of cell number, protein, or RNA. Moreover, the prolonged impacts were assessed at the lower level of adlay (50 µg/ml; Figures 4–7). Consistent with responses in HT-29 cells, DON-induced IL-8 was also significantly reduced by adlay in other colonic epithelial cell lines, such as HCT-8, LS174T, DLD-1, and SW480 (Figure 1D). In addition to these colorectal adenocarcinoma-derived enterocytes, the similar responses in IL-8 expression were confirmed in the well-established non-tumorigenic normal enterocytes (IPEC-1; Figure 1D).

### MAPK and Egr1-Linked Signaling Pathways Are Involved in Adlay-Induced Regulation of IL-8 Expression

**Figure 2:**
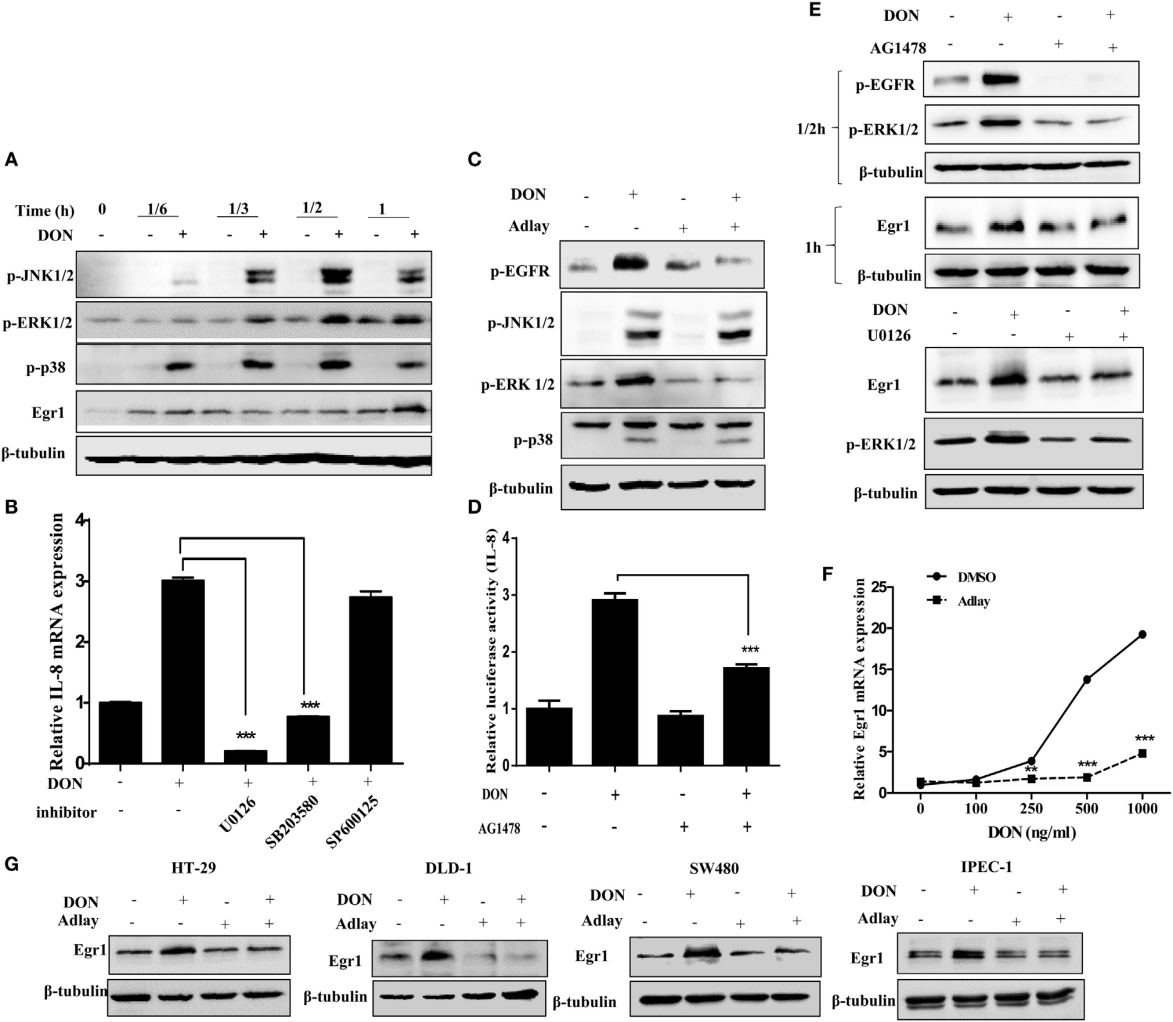
Mitogen-activated protein kinase and Egr1 signaling pathways are involved in adlay-induced regulation of interleukin (IL)-8 expression. **(A)** HT-29 cells were treated with 500 ng/ml deoxynivalenol (DON) for each indicated time, and cell lysates were subjected to Western blot analysis. **(B)** HT-29 cells were pretreated with 5 µM U0126, 5 µM SB203580, or 10 µM SP600125 for 2 h, then exposed to 500 ng/ml DON for another 1 h, and the IL-8 mRNA expression was measured by real-time quantitative polymerase chain reaction (qRT-PCR; *n* = 3, **p < 0.01, ***p < 0.001, significant difference from the DON-treated group without an inhibitor). **(C)** HT-29 cells were treated with 500 ng/ml DON in the absence or presence of 1,000 µg/ml adlay bran extract for 1 h, after which cell lysates were subjected to Western blot analysis. **(D)** HT-29 cells were transfected with wild-type IL-8 promoter-linked reporter plasmid and then treated with 500 ng/ml DON in the absence or presence of 10 µM AG1478 for 12 h before luciferase activity assay (*n* = 3, ***p < 0.001, significant difference from DON-treated group without an inhibitor). **(E)** HT-29 cells were pretreated with 10 µM AG1478 for 2 h, then exposed to 500 ng/ml DON for another 0.5 or 1 h, and the cell lysates were subjected to Western blot analysis (the upper panel). HT-29 cells pretreated with 5 µM U0126 for 2 h were exposed to 500 ng/ml DON for another 1 h, after which cell lysates were subjected to Western blot analysis (the lower panel). **(F)** HT-29 cells were treated with an indicated dose (0, 100, 250, 500, or 1,000 ng/ml) of DON in the absence or presence of 1,000 µg/ml adlay bran extract for 1 h, after which the Egr1 expression was measured using qRT-PCR (*n* = 3, **p < 0.01, ***p < 0.001, significant difference from DMSO-treated group at each dose of DON). **(G)** HT-29, DLD-1, SW480, or IPEC-1 cells were treated with 500 ng/ml DON in the absence or presence of 1,000 µg/ml adlay bran extract for 1 h, after which Egr1 expression was measured by Western blot analysis. All blots are representative of three independent experiments.

As a pivotal transcriptional regulator in DON-induced IL-8 production, Egr1 and MAPKs, which induce DON-induced Egr1 expression in IECs (43), were assessed in this study. As expected, DON treatment activated MAPK signals along with Egr1 induction in HT-29 cells (Figure 2A). Among these DON-elevated signaling mediators, extracellular signal-regulated protein kinases 1 and 2 (ERK1/2) and p38 signals were positively associated with IL-8 mRNA expression in IECs (Figure 2B). In particular, ERK1/2 as a crucial mediator to regulate various physiological functions is activated by receptor tyrosine kinase-associated pathways such as epidermal growth factor receptor (EGFR) (43–45). In the present model, phosphorylation of EGFR and ERK1/2 were downregulated by adlay treatment; however, the JNK1/2 and p38 signals were not notably involved in this regulation (Figure 2C). Moreover, blocking of the EGFR-linked signal significantly downregulated DON-induced IL-8 transcriptional activation (Figure 2D). In terms of signaling pathways, inhibition of EGFR and ERK1/2 signals suppressed the DON-induced expression of Egr1 (Figure 2E), which is a key transcriptional modulator of pro-inflammatory IL-8 expression in IECs as mentioned in Section “Introduction” (Figure S2 in Supplementary Material). Furthermore, DON-induced Egr1 transcription was significantly attenuated in adlay-treated enterocytes (Figure 2F). Suppression of Egr1 levels by adlay treatment was consistently observed in other IECs, including DLD-1, SW480, and IPEC-1 cells (Figure 2G). Taken together, adlay-suppressed IL-8 expression was caused by signaling interference with Egr1 in EGFR- and ERK1/2-dependent manners in IECs.

### Adlay-Enhanced PKC-Mediated Cytoplasmic Translocation of HuR Protein in Response to DON Insult

**Figure 3:**
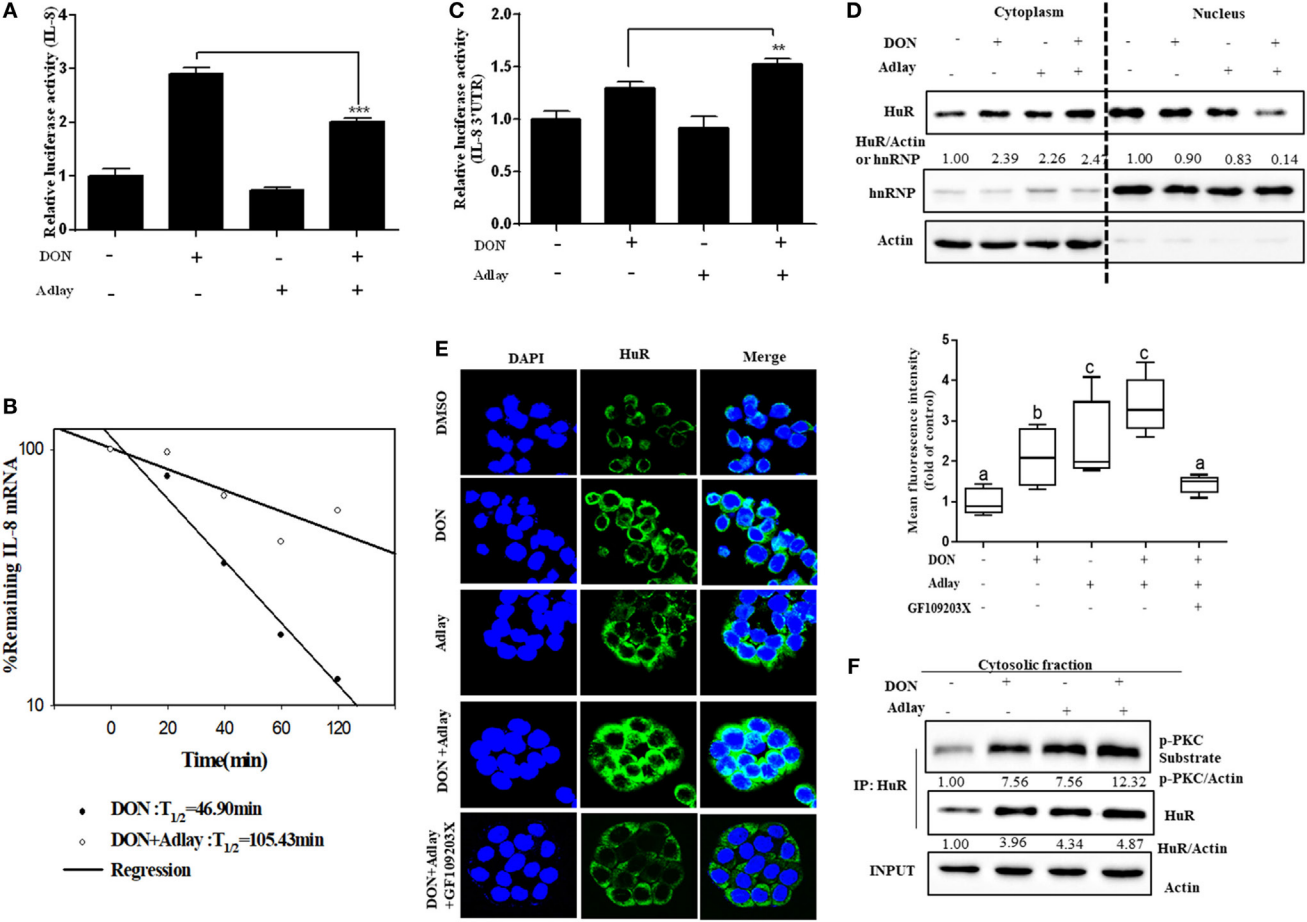
Adlay bran suppress interleukin (IL)-8 transcription despite IL-8 mRNA stabilization *via* human antigen R (HuR). **(A)** HT-29 cells transfected with wild-type IL-8 promoter-linked reporter plasmid were treated with 500 ng/ml deoxynivalenol (DON) in the absence or presence of 250 µg/ml adlay bran extract for 12 h before measurement of the luciferase activity (*n* = 3, ***p < 0.001, significant difference from DON-treated group without adlay). **(B)** HT-29 cells were treated with 500 ng/ml DON in the absence or presence of 250 µg/ml adlay bran extract for 1 h, after which cellular transcription was arrested by adding 5 µM actinomycin D. The remaining IL-8 mRNA at the indicated time point was then quantified by real-time quantitative polymerase chain reaction (*n* = 3, ***p < 0.001). **(C)** HT-29 cells were transiently transfected with a reporter plasmid containing IL-8 3′ untranslated region (UTR) and then treated with 500 ng/ml DON in the absence or presence of 250 µg/ml adlay bran extract for 12 h before measurement of luciferase activity (*n* = 3, **p < 0.01, significant difference from DON-treated group without adlay). **(D)** HT-29 cells were treated with 500 ng/ml DON in the absence or presence of 1,000 µg/ml adlay bran extract for 1 h. HuR protein expressions in cytosolic and nuclear fractions were assessed using Western blot analysis. **(E)** HT-29 cells were treated with 500 ng/ml DON and/or 1,000 µg/ml adlay bran extract in the absence or presence of 5 µM GF109203X for 1 h. Cytosolic or nuclear HuR protein was visualized with anti-HuR antibody (green) or DAPI (blue) staining (the left panel, magnification, ×1,600). The mean fluorescence intensity of cytosolic HuR was quantified [the right graph, different letters **(A–C)** over each column represent a significant difference between two groups, *p* < 0.05]. **(F)** Co-immunoprecipitation (IP) for cytoplasmic extracts of HT-29 cells treated with 500 ng/ml DON in the absence or presence of 1,000 µg/ml adlay bran extract for 1 h. Data are representative of three independent experiments.

Although DON-induced IL-8 transcription was suppressed by adlay exposure (Figure 3A), its mRNA stability was elevated by treatment with adlay in HT-29 cells (Figure 3B). Moreover, we constructed a luciferase reporter system containing 3′UTRs of IL-8, which have 10 AU-rich elements (AREs). DON-induced luciferase activity was also enhanced by adlay treatment in IECs (Figure 3C), indicating the elevated stability of DON-induced IL-8 mRNA by adlay. Although most RNA-binding proteins destabilize targeted mRNA, a few of these proteins, including HuR, bind to 3′UTRs with AREs and stabilize the transcripts of genes, including those encoding growth factors or pro-inflammatory cytokines (36, 46). Consistent with the promoting effects of adlay on IL-8 mRNA stability, adlay exposure enhanced the cytoplasmic translocation of HuR protein triggered by DON in enterocytes (Figure 3D). These patterns were also confirmed by visualizing nuclear localization of HuR protein in the cells by confocal microscopy (Figure 3E). In terms of the signaling pathway, PKC was evaluated for its involvement in HuR translocation to the cytoplasm (47, 48). Moreover, adlay-enhanced cytoplasmic translocation of HuR protein was significantly suppressed by PKC inhibition (Figure 3E). In addition, samples were analyzed to determine whether PKC is involved in regulation of HuR protein in adlay-exposed IECs. Adlay extract enhanced DON-induced HuR protein translocation to the cytoplasm *via* PKC-mediated phosphorylation in IECs (Figure 3F). Taken together, adlay-enhanced PKC-mediated cytoplasmic translocation of the HuR protein, although adlay treatment decreased DON-induced IL-8 production at the transcriptional level.

### Adlay Counteracts Epithelial Barrier Disruption by DON in PKC- and HuR-Linked Ways

**Figure 4:**
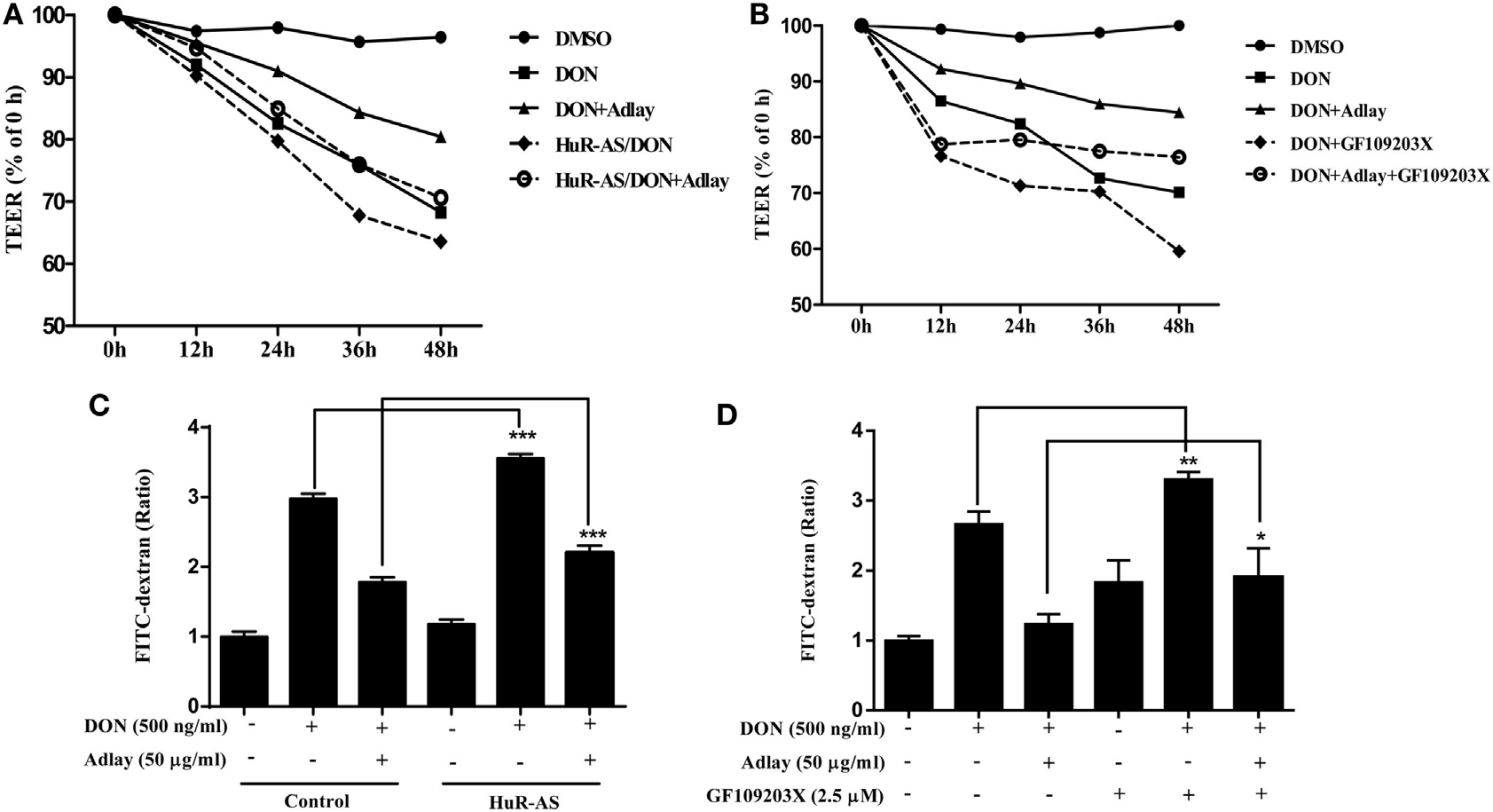
Treatment with adlay bran attenuates deoxynivalenol (DON)-induced barrier disruption in a protein kinase C/human antigen R (HuR)-linked pathway. Epithelial barrier function was determined by the measurement of transepithelial electrical resistance (TEER) **(A,B)** or translocation of fluorescein isothiocyanate-dextran (FITC)–dextran to the basolateral compartment **(C,D)**. **(A)** Control or HuR antisense (HuR-AS)-expressing HCT-8 cells monolayer was exposed to 500 ng/ml DON in the absence or presence of 50 µg/ml adlay bran extract for the indicated time. **(B)** HCT-8 cells monolayer was exposed to 500 ng/ml DON and/or 50 µg/ml adlay in the absence or presence of 2.5 µM GF109203X for the indicated time. **(C)** Control or HuR-AS-expressing HCT-8 cells monolayer was exposed to 500 ng/ml DON in the absence or presence of 50 µg/ml adlay bran extract for 48 h. **(D)** HCT-8 cells monolayer was exposed to 500 ng/ml DON and/or 50 µg/ml adlay in the absence or presence of 2.5 µM GF109203X for 48 h (*n* = 3, *p < 0.05, **p < 0.01, and ***p < 0.001, significant difference from DON-treated group with or without adlay). Data are representative of three independent experiments.

In addition to epithelial chemokine production, we assessed the effects of adlay on intestinal barrier integrity by measuring the TEER *in vitro*. The DON-disrupted intestinal barrier was partly restored by adlay extract treatment; however, HuR suppression and PKC inhibition attenuated the protective actions of adlay against barrier disruption (Figures 4A,B). Another readout of the intestinal permeability by quantifying the paracellular passage of 4 kDa FITC–dextran indicated a similar pattern of adlay-induced protection against DON-induced epithelial disruption in HuR- and PKC-dependent ways (Figures 4C,D). We next investigated how adlay restored DON-disrupted intestinal barrier integrity through enhanced translocation of the HuR protein.

**Figure 5:**
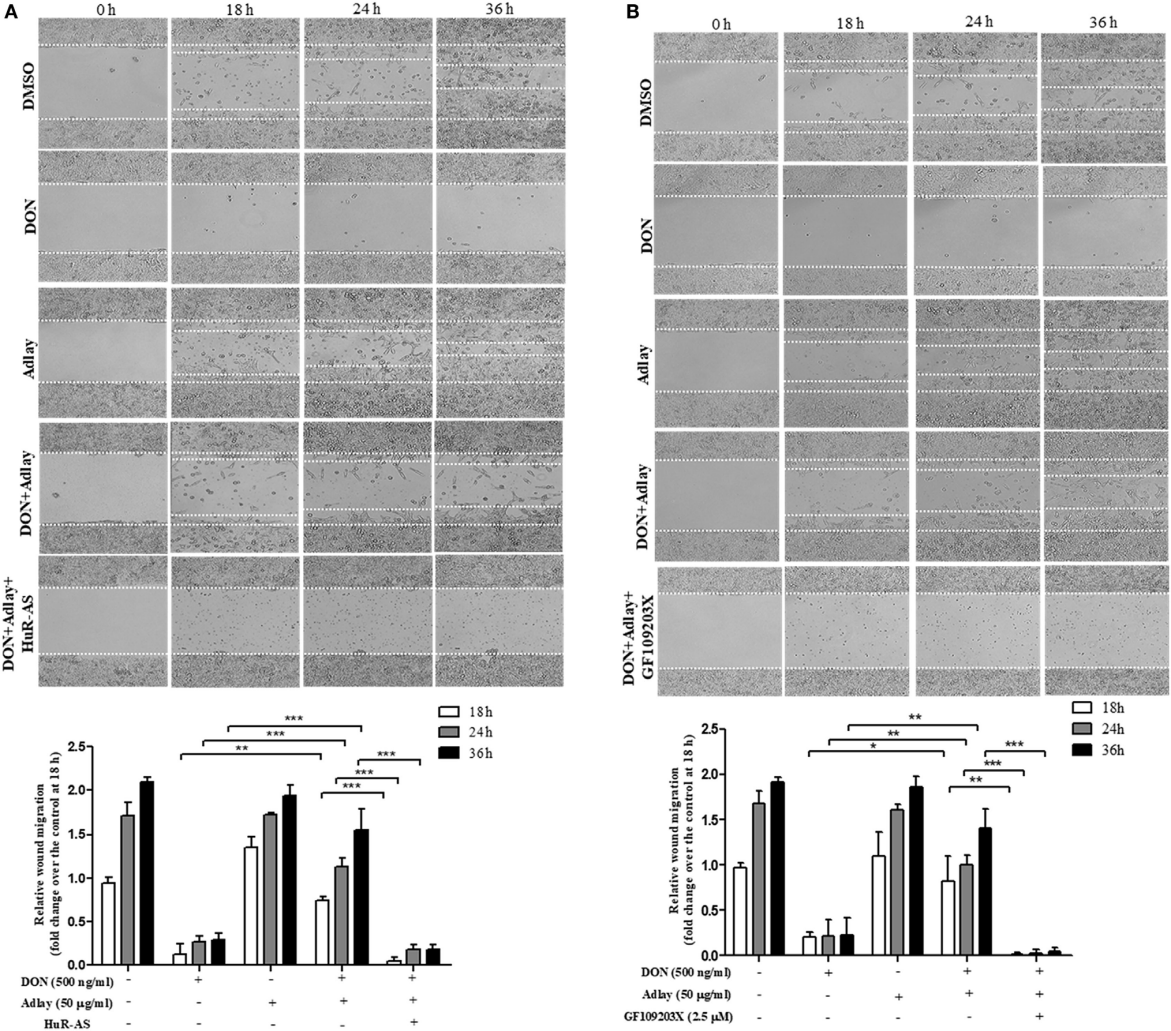
Treatment with adlay bran counteracts deoxynivalenol (DON)-inhibited epithelia migration in a protein kinase C/human antigen R (HuR)-linked pathway.**(A,B)** Control or HuR antisense (HuR-AS)-expressing HCT-8 cells monolayer was wounded by ibidi culture-Insert, and the epithelial migration was then measured at 0, 18, 24, and 36 h after treatment with 500 ng/ml DON and/or 50 µg/ml adlay bran extract in the absence **(A)** or presence **(B)** of 2.5 µM GF109203X (original magnification, ×100 under the phase-contrast microscope). The relative migration was quantified by measuring the migration length from the initial wounded edges (*n* = 3, *p < 0.05, **p < 0.01, and ***p < 0.001).

To explore epithelial barrier restitution by cellular migration, we assessed wound healing of migrating enterocytes exposed to DON and adlay. DON exposure retarded the migration activities in response to physical wounds in enterocytes (Figure 5). In contrast, treatment with adlay significantly restored the epithelial restitution in DON-insulted cells, which was attenuated by inhibition of HuR expression and PKC activity in IECs (Figures 5A,B), indicating that DON-disrupted intestinal barrier integrity was rescued by adlay extract exposure *via* PKC- and HuR protein-mediated enhancement of migration of insulted IECs. The PKC- and HuR-linked signals were also positively involved in the scratch-induced wound healing process in the epithelial monolayer (Figures S3A,B in Supplementary Material, respectively).

### Treatment with Adlay Regulates DON-Induced Cytoskeleton Rearrangement in a Ras Homolog Gene Family Member A (RhoA) GTPase-Dependent Manner

**Figure 6:**
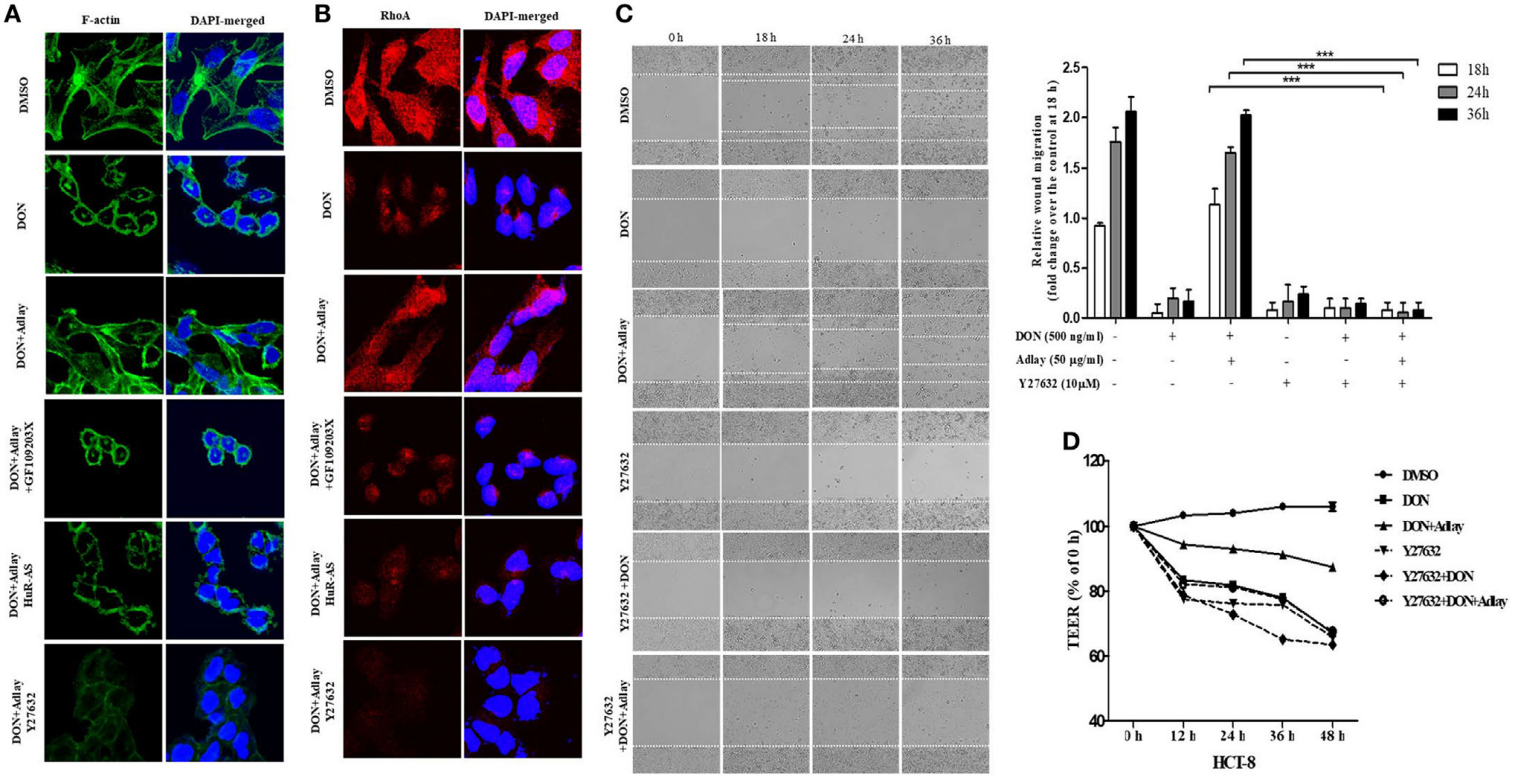
Treatment with adlay bran counteracts deoxynivalenol (DON)-induced cytoskeleton rearrangement in a Ras homolog gene family member A (RhoA) GTPase-dependent manner. **(A,B)** Control or human antigen R antisense (HuR-AS)-expressing HCT-8 cells were co-treated with 500 ng/ml DON and/or 50 µg/ml adlay bran extract in the absence or presence of 2.5 µM GF109203X or 10 µM Y27632 for 24 h. **(A)** F-actin staining by fluorescein isothiocyanate–phalloidin (green), **(B)** RhoA protein (red) or DAPI (blue) were visualized. The representative figures using confocal microscopic observation are shown at 2,400× magnification. Data are representative of three independent experiments. **(C)** Wounded HCT-8 cell monolayer was treated with 500 ng/ml DON and/or 50 µg/ml adlay bran extract in the absence or presence of 10 µM Y27632. Images of the cells migrating into the wound area were captured at 0, 18, 24, and 36 h by phase-contrast microscopy (original magnification, ×100). The relative migration was quantified by measuring the migration length from the initial wounded edges (*n* = 3, ***p < 0.001, significant difference from DON/adlay-treated group without an inhibitor). **(D)** Epithelial barrier function was determined by measurement of transepithelial electrical resistance (TEER). HCT-8 cells monolayer was exposed to 500 ng/ml DON and/or 50 µg/ml adlay in the absence or presence of 10 µM Y27632 for the indicated time. All of the results are representative of three independent experiments.

**Figure 7:**
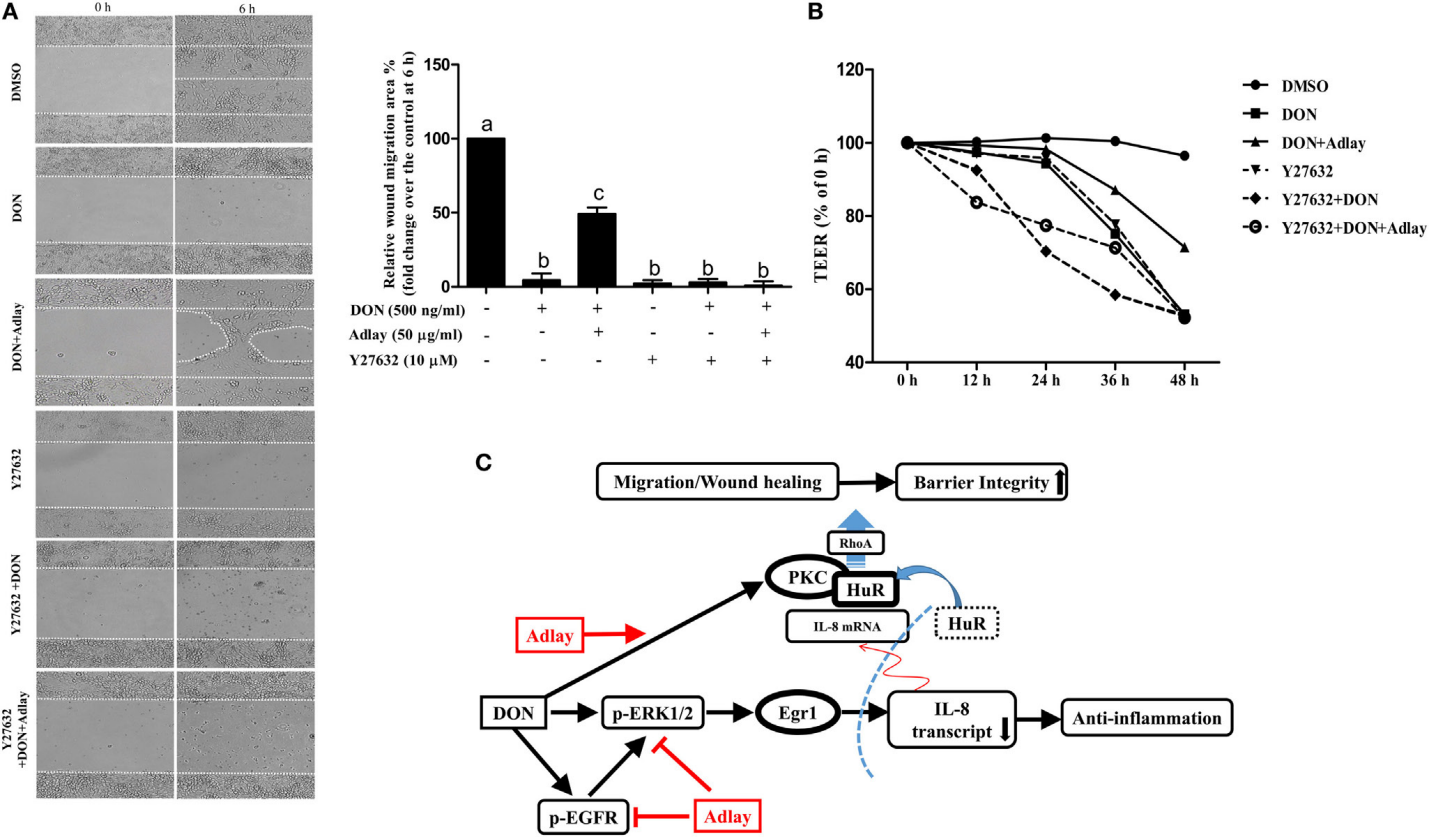
Treatment with adlay bran counteracts deoxynivalenol (DON)-inhibited IPEC-1 cell migration and epithelial barrier in a Ras homolog gene family member A (RhoA) GTPase-dependent manner. **(A)** Wounded IPEC-1 cell monolayer was treated with 500 ng/ml DON and/or 50 µg/ml adlay bran extract in the absence or presence of 10 µM Y27632. Images of the cells migrating into the wound area were captured at 0 and 6 h and after treatment by phase-contrast microscopy (original magnification, ×100). The relative migration was quantified by measuring the migration area from the initial wounded edges. Different letters **(A–C)** over each *column* represent a significant difference between two groups (*p* < 0.05). **(B)** Epithelial barrier function was determined by the measurement of transepithelial electrical resistance (TEER) assay. IPEC cells monolayer was exposed to 500 ng/ml DON and/or 50 µg/ml adlay in the absence or presence of 10 µM Y27632 for the indicated time. Data are representative of three independent experiments. **(C)** Schematic network of adlay-induced protection against DON-induced inflammation and epithelia barrier injury. In the schematic signaling pattern, adlay suppresses DON-induced pro-inflammatory chemokine production by reducing IL-8 transcription in a mitogen-activated protein kinase/Egr1-dependent way, despite upregulation of the mRNA stabilization *via* human antigen R (HuR). Instead, protein kinase C (PKC)-activated cytoplasmic HuR plays critical roles in the intestinal epithelial restitution and maintenance of barrier integrity by modulating RhoA-mediated cytoskeletal rearrangement under stress of DON.

Migrating HCT-8 enterocytes were closely observed after staining actin polymerization. DON treatment notably suppressed the staining of F-actin formation with lamellipodia and filopodia networks (Figure 6A) but was retarded by adlay exposure. In contrast, adlay-induced resistance to cytoskeletal rearrangement in the gut epithelial cells was notably mediated in PKC- and HuR-dependent manners (Figure 6A). Among the critical signaling mediators of lamellipodia or filopodia formation, RhoA GTPase was quantified in the present enterocyte exposure model. In agreement with the changes in F-actin formation, DON treatment suppressed the RhoA levels, which was counteracted by the adlay treatment. Adlay-induced restoration of the actin cytoskeleton network was dependent on PKC activation and HuR protein (Figure 6B). Functionally, inhibition of RhoA GTPase activities significantly suppressed adlay-restored enterocyte migration under the insult of enteropathogenic DON (Figure 6C). Assessment in the scratch-induced wound healing consistently confirms the positive involvement of RhoA signals in restoring of epithelial migration in the presence of DON and adlay (Figure S3C in Supplementary Material). Ultimately, inhibition of RhoA GTPase activities significantly suppressed adlay-restored epithelial barrier under the insult of DON in HCT-8 cells (Figure 6D). Finally, mechanistic implication was also tested using the non-tumorigenic enterocyte IPEC-1. Inhibition of RhoA GTPase activities significantly suppressed adlay-restored enterocyte migration in the presence of DON (Figure 7A). Furthermore, RhoA signal was positively involved in maintaining the adlay-restored epithelial barrier under the stress of DON in IPEC-1-based monolayer (Figure 7B). Taken together, RhoA GTPase-linked signal promotes cell migration *via* cytoskeletal rearrangement, which contributes to the epithelial restitution in the insulted gut barrier. Therefore, it can be concluded that adlay counteracts DON-induced migratory inhibition by restoring cytoskeletal rearrangement and RhoA expression in the gut epithelial cells in HuR and PKC-dependent pathways (Figure 7C).

## Discussion

Although adlay can be contaminated with DON, components of adlay bran were shown to attenuate the toxin-induced adverse effects in enterocytes. This potent gastrointestinal crosstalk was caused by a reduction in pro-inflammatory chemokines and increased epithelial restitution in the presence of adlay components, which would reduce mold-related risks such as gut inflammation and ulcerative injuries after human consumption (Figure 7C). In the present enterocyte model, adlay treatment provides a way of protecting against DON-induced gastrointestinal barrier disruption. The protective actions of adlay against gastrointestinal illness can be speculated with their anti-inflammatory and antioxidative properties (30, 35, 49). In particular, 15 compounds, including a novel aurone derivative, two chromones, one dihydrochalcone, one chalcone, four flavanones, five flavones, and one isoflavone, were purified from the anti-inflammation-guided fractionation (30). Ethyl acetate fraction has been the potential pool of anti-inflammatory components such as eriodictyol and the ceramide (2S,3S,4R)-2-[(2’R)-2’-hydroxytetracosanoyl-amino]-1,3,4-octadecanetriol (35). Moreover, analysis of the phenolic compounds identified ferulic acid as the major anti-inflammatory component in the ethyl acetate fraction (35). Ferulic acid is also an effective chemopreventive component in adlay bran against tumorigenic processes of the colon cancer (29, 50). These dietary phytochemicals are metabolized into active agents affecting gut health and alter the composition of gut microbiota (51, 52). Of note, cecal and fecal levels of total short-chain fatty acid including butyric acid are significantly elevated by adlay consumptions (52), suggesting alterations in the growth of intestinal bacteria and production of beneficial microbial metabolites for the gastrointestinal physiology and health.

Mechanistically, NF-κB has been shown to be a well-known target of anti-inflammatory actions of adlay (31, 53). However, the intestinal regulation of inflammatory signals by adlay was not mediated by the canonical pro-inflammatory NF-κB-linked pathway. Instead, adlay regulated Egr1, a key transcription factor, which is a crucial player in DON-induced inflammatory stimulation in enterocytes. In the porcine model, DON also enhanced Egr1 expression while suppressing expression and phosphorylation of p65, a crucial subunit of pro-inflammatory NF-κB (54). Among the MAPKs in other models, DON-activated ERK1/2 also mediates epithelial Egr1 induction (43, 54), and ERK1/2 inhibition by adlay leads to suppression of DON-induced pro-inflammatory chemokine production (Figure 7C). Adlay downregulated ERK1/2- and Egr1-mediated transcriptional activation, which was sufficient to attenuate IL-8 production although adlay bran enhanced the stability of IL-8 transcript *via* HuR protein. This paradoxical elevation of mRNA stability was due to increased translocation of the HuR protein to the cytoplasm by adlay exposure. In this study, HuR protein was more important at maintaining the epithelial barrier integrity than the epithelial inflammatory responses to mucosal DON. It has been reported that DON induces the intestinal barrier disruption partially through early activation of the MAPK p44/42 pathway (55). Our results showed the involvement of PKC signaling in adlay-induced protective action against DON-induced barrier disruption since PKC inhibition decreased epithelial restitution and subsequent epithelial barrier permeability of the enterocyte monolayer in the presence of adlay. Moreover, adlay-activated PKC promoted the cytoplasmic translocation of HuR protein, which contributed to enhanced barrier integrity under the insult by the enteropathogenic mycotoxin and increased the permeability, suggesting that HuR is also positively involved in adlay-induced recovery of barrier integrity. Adlay extracts alone have no effects on epithelial cell migration, whereas it counteracted DON-induced impairment of the epithelial barrier, emphasizing its protective capacity against toxic injuries in the gut. The reduced epithelial motility (restitution) by DON exposure can be associated with significant remodeling of the actin cytoskeleton, as well as changes in the expression of adhesion or connecting molecules such as focal adhesion kinase and connexin-43 (56). HuR-linked wound healing or maintenance of the barrier integrity has also been reported, primarily through its actions on cellular adhesion (57, 58). Therefore, it can be speculated that adlay coexistence improves epithelial migration and wound restitution by repressing disruption of the cytoskeletons and its machineries in PKC-HuR-dependent manners although the anti-inflammatory actions are due to EGFR-ERK1/2-Egr1-asssociated transcriptional activation rather than the circuit of PKC-HuR protein in response to gastrointestinal insult by DON.

According to a recent *in vivo* study, exposure to 2.3 mg DON/kg diet for 35 days corresponds to 7.7 µM DON in serum levels, which induces significant histopathological changes and stress signaling activation in the pig intestine (59, 60). However, this exposure level (2.3 mg DON/kg feed) is not frequent in cereals used for human food and animal feed. The range of DON concentrations plausibly encountered in the gastrointestinal tract after consumption of DON contaminated food can be derived from exposure assessment based on estimated daily intakes through the Scientific Co-operation on Questions relating to Food (SCOOP) program (61). The DON concentration (160 ng/ml) and higher DON concentration (2,000 ng/ml) correspond to the estimated daily chronic exposure to mean levels of DON among adults and the exposure levels after consuming a diet contaminated with a high level of DON among children, respectively (62, 63). Moreover, according to the recent survey of 7,049 feed samples sourced in North and South America, Europe, and Asia during 2009–2011, 59% of raw feedstuffs and compound feed samples were contaminated with DON, with an average contamination level of 1 mg DON/kg feed, corresponding to about 992 ng/ml DON in serum levels (64). Therefore, in our model, we used 100–1,000 ng/ml DON, which corresponds to *in vivo* exposure levels (about 0.1–1.0 mg DON/kg food/feed), which is assumed to be realistic within the average contamination level in both food and feed. According to our previous investigation, although the DON contamination levels in total adlay were 0.37–2.55 mg/kg, the bran levels were around 0.4–1.0 mg/kg adlay (5). Although high levels of trichothecene mycotoxins such as nivalenol and DON (>2,000 ng/ml) significantly influence enterocyte viability through activation of pro-apoptotic processes, ingestion of the realistic milder levels of DON (<1,000 ng/ml) retarded the epithelial cell migration (restitution), representing the initial step in the wound healing process in the gastrointestinal tract.

In conclusion, adlay components reduced gastrointestinal injuries by adlay-contaminating DON in enterocyte models. This potent gut protective crosstalk was associated with a reduction in pro-inflammatory chemokines and increased epithelial restitution in the presence of adlay components. Mechanistically, adlay regulated ERK1/2 and Egr1, a key transcription factor, which are crucial players in DON-induced inflammatory stimulation in enterocytes. Moreover, adlay improved the gut epithelial barrier by attenuating DON-induced migratory inhibition *via* cytoskeletal rearrangement in RhoA-dependent manner in the gut epithelial cells. All of the mechanistic implications using the *in vitro* models may account for the adlay-induced reduction of enteropathogenic insults including gut inflammation and ulcerative injuries following human consumption.

## Author Contributions

Project design and hypotheses were made by YM and ZD. ZD, KHK and JK conducted experiments and analyzed data. YM and ZD prepared the manuscript. YM supervised the overall project.

## Conflict of Interest Statement

The authors declare that the research was conducted in the absence of any commercial or financial relationships that could be construed as a potential conflict of interest. The reviewers IM, SA, and handling editor declared their shared affiliation.
