# Supplementary material for: Fungal Deoxynivalenol-Induced Enterocyte Distress Is Attenuated by Adulterated Adlay: In Vitro Evidences for Mucoactive Counteraction
Source: Front Immunol. 2018 Feb 23;9:186. doi: 10.3389/fimmu.2018.00186 (PMC5829524; doi:10.3389/fimmu.2018.00186)
Supplement: Supplementary file 1 [file Data_Sheet_1.PDF]

Supplementary Material

Fungal Deoxynivalenol-induced Enterocyte Distress is Attenuated by Adulterated Adlay: *In vitro* Evidences for Mucoactive Counteraction

Zhimin Du[1], Juil Kim[1] and Yuseok Moon[1,2,*]

*** Correspondence:** Yuseok Moon: moon@pnu.edu

1.1 Supplementary Figures

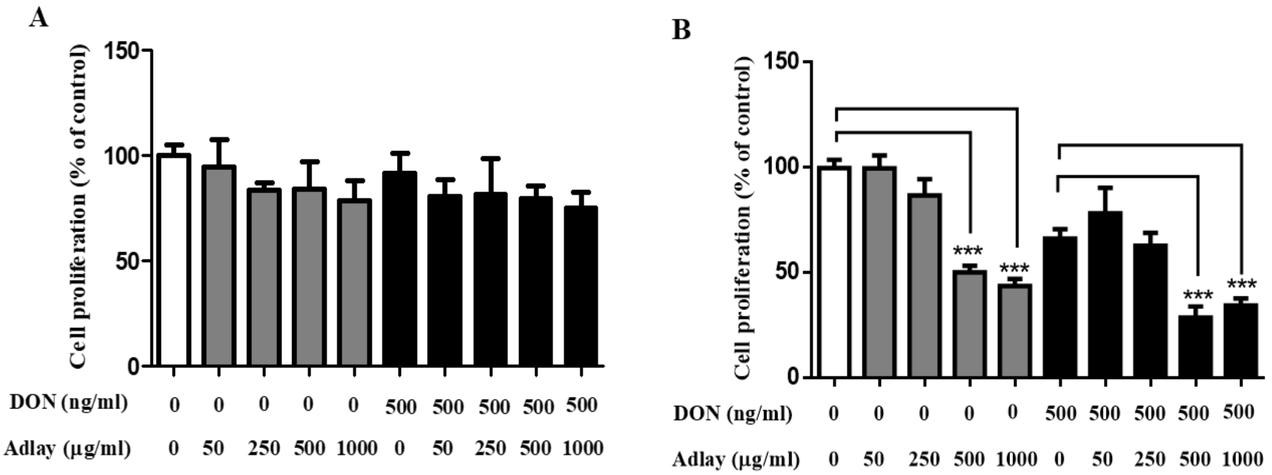

Figure S1. Effects of adlay and DON on the cell proliferation. HT-29 cells were treated with the indicated dose (0, 50, 250, 500 or 1000 µg/ml) of adlay bran extract in the absence or presence of 500 ng/ml DON for 1 h (A) or 24 h (B) (n=6,*** p <0.001, significant difference from vehicle- or DON-treated group without adlay).

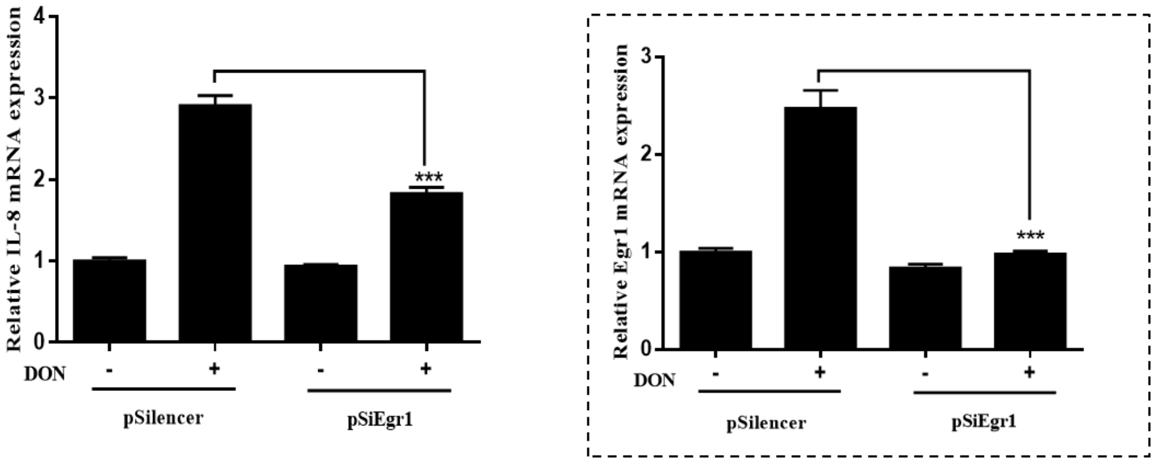

Figure S2. Effects of adlay on DON-induced IL-8 expression. HT-29 cells transfected with the empty vector (control) or shEgr1 were treated with vehicle or 500 ng/ml DON for 1 h. Each mRNA expression was assessed by qRT-PCR. The graph in the box indicated the efficient suppression of Egr1 levels by its shRNA (n=3,*** $p < 0.001$, significant difference from DON-treated pSilencer-transfected group). All of the results are representative of three independent experiments.

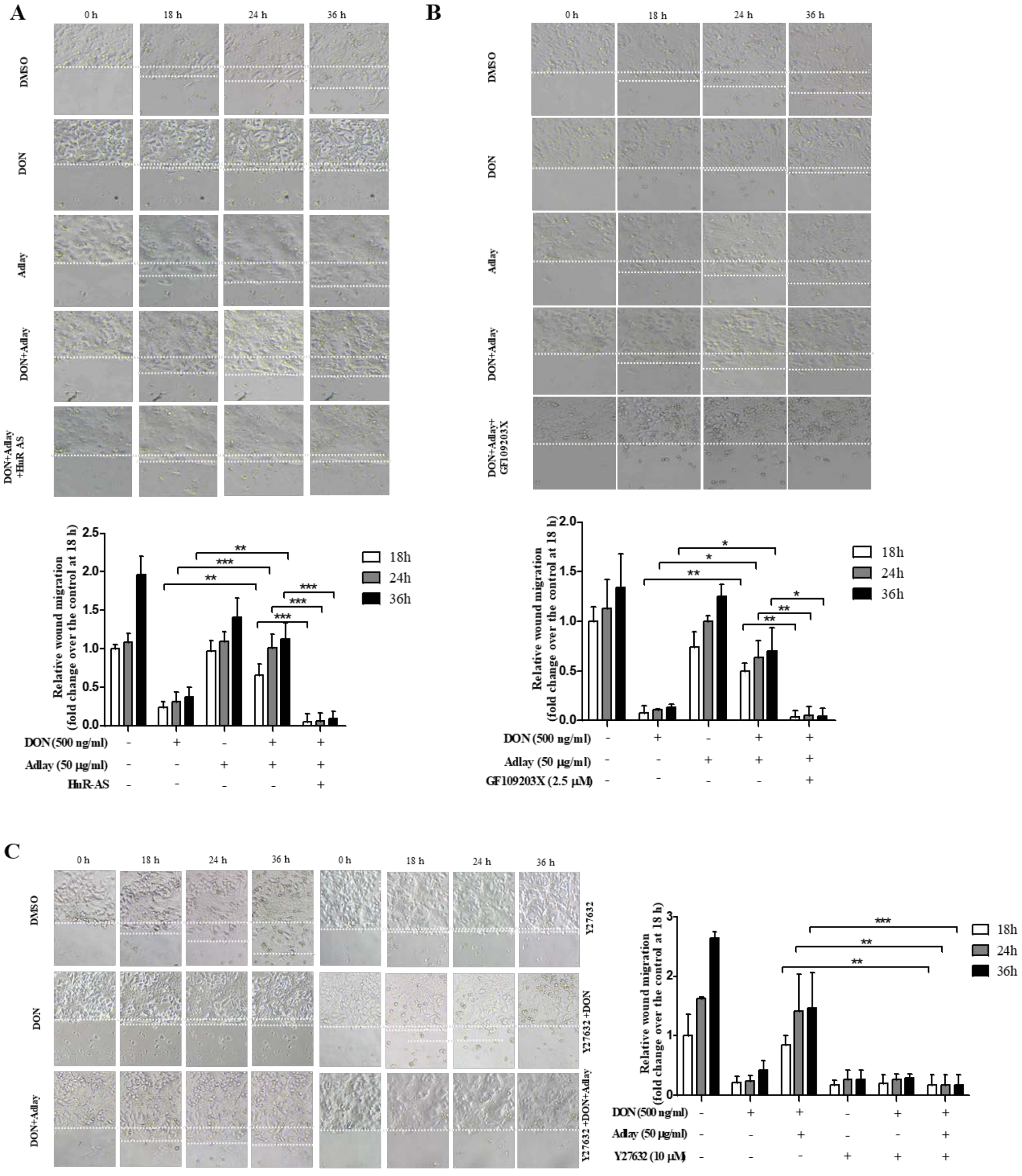

Figure S3. Treatment with adlay counteracts DON-inhibited epithelia migration in a PKC/HuR-linked pathway. (A–B) Control or HuR antisense (HuR-AS)-expressing HCT-8 cells monolayer was wounded by physical scratching, and the epithelial restitution was then measured at 0 h, 18 h, 24 h and 36 after treatment with 500 ng/ml DON and/or 50 µg/ml adlay bran extract in the absence (A) or presence (B) of 2.5 µM GF109203X. (C) Wounded HCT-8 cell monolayer or was treated with 500 ng/ml DON and/or 50 µg/ml adlay bran extract in the absence or presence of 10 µM Y27632. Images of the cells migrating into the wound area were captured at 0 h, 18 h, 24 h and 36 h by phase contrast

microscopy (original magnification × 100 under the phase-contrast microscope). The relative migration was quantified by measuring the migration length from the scratch edges (n=3-6,* $p < 0.05$, ** $p < 0.01$, and *** $p < 0.001$, significant difference from DON/adlay-treated group without an inhibitor). All of the results are representative of three independent experiments.
